# Contrasting Evolutionary Patterns of Functional Connectivity in Sensorimotor and Cognitive Regions after Stroke

**DOI:** 10.3389/fnbeh.2016.00072

**Published:** 2016-04-11

**Authors:** Huaigui Liu, Tian Tian, Wen Qin, Kuncheng Li, Chunshui Yu

**Affiliations:** ^1^Department of Radiology and Tianjin Key Laboratory of Functional Imaging, Tianjin Medical University General HospitalTianjin, China; ^2^Department of Radiology, Xuanwu Hospital of Capital Medical UniversityBeijing, China

**Keywords:** cerebral infarction, functional neuroimaging, magnetic resonance imaging, motor cortex, neuronal plasticity

## Abstract

The human brain is a highly connected and integrated system. Local stroke lesions can evoke reorganization in multiple functional networks. However, the temporally-evolving patterns in different functional networks after stroke remain unclear. Here, we aimed to investigate the dynamic evolutionary patterns of functional connectivity density (FCD) and strength (FCS) of the brain after subcortical stroke involving in the motor pathways. Eight male patients with left subcortical infarctions were longitudinally examined at five time points within a year. Voxel-wise FCD analysis was used to identify brain regions with significant dynamic changes. The temporally-evolving patterns in FCD and FCS in these regions were analyzed by a mixed-effects model. Associations between these measures and clinical variables were also explored in stroke patients. Voxel-wise analysis revealed dynamic FCD changes only in the sensorimotor and cognitive regions after stroke. FCD and FCS in the sensorimotor regions decreased initially, as compared to controls, remaining at lower levels for months, and finally returned to normal levels. In contrast, FCD and FCS in the cognitive regions increased initially, remaining at higher levels for months, and finally returned to normal levels. Most of these measures were correlated with patients’ motor scores. These findings suggest a network-specific dynamic functional reorganization after stroke. Besides the sensorimotor regions, the spared cognitive regions may also play an important role in stroke recovery.

## Introduction

Motor pathways are frequently impaired in stroke patients with subcortical infarction. In most of these patients, the impaired motor function recovers in the first several months after stroke (Kwakkel et al., 2004), and this recovery has been attributed to a normalization of activity (Ward et al., 2003; Tombari et al., 2004; Kim et al., 2006) and connectivity (Golestani et al., 2013; Rehme and Grefkes, 2013) in the sensorimotor network (SMN). The human brain is composed of multiple highly connected and integrated functional networks. If a network is damaged, other networks may reorganize themselves to facilitate the functional recovery of the damaged network. This hypothesis is supported by findings of increased connectivity in several non-sensorimotor networks in patients with subcortical stroke (Wang et al., 2014). However, the dynamic connectivity changes of non-sensorimotor networks after subcortical stroke remain largely unknown.

In stroke patients, most resting-state functional connectivity studies are based on *a priori* selection of seed regions (Park et al., 2011; Xu et al., 2014), which cannot provide a full picture of connectivity changes in the whole brain. Moreover, previous studies only focused on functional connectivity strength (FCS) changes between brain regions, leaving post-stroke functional connectivity density (FCD) changes largely unknown. The FCD mapping is a newly developed data-driven method that measures the connectivity density of each voxel (Tomasi and Volkow, 2010). It is a plausible method to identify connectivity changes in the range of the whole brain.

In this study, we adopted a longitudinal design to investigate post-stroke connectivity changes and associations of these changes with motor recovery. First, we performed a voxel-wise FCD analysis to identify brain regions exhibiting longitudinal connectivity changes after subcortical infarctions involving the motor pathways. Second, we investigated post-stroke temporally-evolving patterns in FCD and functional connectivity strength (FCS) in these hub regions. Finally, we explored associations of these altered connectivity properties with clinical outcomes in stroke patients. We hypothesize that some non-SMN regions would also display longitudinal post-stroke connectivity changes based on clues from a cross-sectional study (Wang et al., 2014). We further hypothesize that the SMN and non-SMN regions would exhibit different evolutionary patterns following stroke.

## Materials and Methods

### Subjects

The Ethics Committee of Xuanwu Hospital approved this experiment, and each participant gave written informed consent. This study included eight right-handed male patients (mean age: 49.0 years, range: 41–55 years) with first-onset subcortical infarctions in the left internal capsule and neighboring regions involving the motor pathways. None of the patients had a history of prior neurological or psychiatric disorders, and the patients did not experience any subsequent symptomatic stroke during the time period of the study. The motor function was assessed using the Motricity Index (MI; Demeurisse et al., 1980). This scale measures motor abilities including hand grasping, elbow flexion, shoulder abduction, ankle dorsiflexion, knee extension and hip flexion in the limbs on the affected side. The global functional impairment was assessed using the National Institutes of Health Stroke Scale (NIHSS). The patients were scanned and clinically assessed at five time points, i.e., within 1 week and at 2 weeks, 1 month, 3 months and 1 year after stroke. The demographic and clinical characteristics of the stroke patients are summarized in Table 1. Ten age- and handedness-matched healthy male subjects (mean age: 49.4 years, range: 42–55 years) were recruited for comparisons.

**Table 1:** **Demographic and clinical data for stroke patients**.

Patient number	1	2	3	4	5	6	7	8
Age (years)	42	48	53	52	51	50	55	41
Sex	M	M	M	M	M	M	M	M
Localization of infarct	IC	IC	IC	IC	IC	IC	IC	IC
	CR	CR	CR	CR	CR	CR		CR
		BG	BG	BG				BG
Past medical history	–	HT	–	HT	HT	HT	HT	DT
		HL					DT	
Number of scans	5	5	5	5	5	4	4	5
Scan time (days post-stroke)	4	1	2	0	4	–	6	4
	13	12	16	14	13	11	12	13
	32	35	34	30	27	33	31	29
	147	88	97	92	93	93	60	111
	354	301	350	369	411	432	–	375
Motricity Index (0–200)	33	0	14	141	14	–	37	0
	88	14	58	183	37	86	53	14
	130	19	88	198	47	138	91	33
	190	82	113	198	88	179	102	78
	190	95	113	198	116	183	–	83
National Institutes of Health Stroke	10	14	8	5	10	–	8	15
Scale (0–15)
	3	11	6	2	8	6	7	13
	2	10	3	2	8	5	5	13
	0	8	2	0	5	2	3	6
	0	5	2	0	2	1	–	6

*BG, basal ganglia; CR, corona radiate; DT, diabetes; HL, hyperlipidemia; HT, hypertension; IC, internal capsule; M, male; “–”, no functional MRI data*.

### Image Data Acquisition

All images were acquired using a Siemens Trio 3.0 Tesla MRI scanner (Siemens, Erlangen, Germany). Tight but comfortable foam padding was used to minimize head motion, and earplugs were used to reduce scanner noise. The functional MRI (fMRI) data were collected using an echo-planar imaging (EPI) sequence with the following parameters: repetition time (TR) = 2000 ms, echo time (TE) = 30 ms, field of view (FOV) = 220 mm × 220 mm, matrix = 64 × 64, flip angle = 90°, slice thickness = 3 mm, gap = 1 mm, 32 interleaved transversal slices, and 180 volumes. During fMRI scans, all subjects were instructed to keep their eyes closed, stay as motionless as possible, and not fall asleep. After the scan, fMRI images and subjects’ conditions were checked to confirm whether they satisfied the requirement. If not, the fMRI data were abandoned and scanned again. Structural images were obtained in a sagittal orientation employing a magnetization-prepared rapid gradient-echo sequence over the whole brain: TR = 1600 ms, TE = 2.6 ms, FOV = 256 mm × 224 mm, matrix = 256 × 224, flip angle = 9°, slice thickness = 1 mm, gap = 0 mm, and 176 slices.

### fMRI Data Preprocessing

Resting-state fMRI data were preprocessed using Statistical Parametric Mapping Software (SPM8)^1^. The first 10 volumes for each subject were discarded to allow the signal to reach equilibrium and the participants to adapt to the scanning noise. The remaining 170 volumes were then corrected for the acquisition time delay between slices. All subjects’ fMRI data were within defined motion thresholds (translational or rotational motion parameters less than 2 mm or 2°). The framewise displacement (FD), which indexes volume-to-volume changes in head position, was also calculated based on the derivatives of the rigid body realignment estimates that were used to realign fMRI data (Power et al., 2012). The average FD was considered a nuisance covariate throughout the imaging analyses. All data were spatially normalized to a standard EPI template and re-sampled into a voxel size of 3 × 3 × 3 mm^3^. After normalization, several nuisance covariates (six motion parameters and average BOLD (blood oxygenation level dependent) signals of the ventricular and white matter) were removed from the data using a regression analysis. Finally, the datasets were band-pass filtered with a frequency range of 0.01–0.08 Hz.

### FCD Calculation

We calculated the FCD of each voxel using an in-house script that was based on a previously described method (Tomasi and Volkow, 2010). FCD calculations were restricted to voxels within gray matter (GM) regions with signals greater than 50% of the mean for the whole brain to minimize unwanted effects from susceptibility-related signal loss. Pearson correlation coefficients were used to calculate the FCS, and a pair of voxels with a correlation coefficient *r* > 0.6 was considered functionally connected. These two thresholds were recommended for FCD calculation (Tomasi and Volkow, 2010). The global FCD of a given voxel (x_0_) was defined as the number of voxels that were functionally connected with voxel x_0_. The short-range FCD of voxel x_0_ was defined as the total number of directly and indirectly neighboring voxels that were functionally connected with x_0_. Specifically, we first calculated the FCS between x_0_ and each voxel (x_i_) that was a direct neighbor of x_0_. For each x_i_, if the FCS was significant (*r* > 0.6), then x_i_ was counted as a neighboring voxel that was functionally connected to x_0_. Then, we calculated the FCS between x_i_ and each voxel (x_j_) that was a direct neighbor of x_i_ but not of x_0_. For each x_j_, if the FCS was significant (*r* > 0.6), then x_j_ was also counted as a neighboring voxel that was functionally connected to x_0_. This search strategy was continued until no further voxels could be included. Long-range FCD was calculated as the global FCD minus the short-range FCD and thus reflected the number of non-neighboring voxels of x_0_ that were functionally connected to x_0_. Thus, the short- and long-range FCDs provide information about the relative rather than the absolute spatial distance between voxels. To increase the normality of the distribution, grand mean scaling of short- and long-range FCDs was performed by dividing the FCD of each voxel by the mean value for the whole brain of each subject. Finally, normalized FCD maps were spatially smoothed with a Gaussian kernel of 8 × 8 × 8 mm^3^ full width at half maximum (FWHM).

### Region of Interest (ROI)-Based FCS Calculation

Brain regions with significant FCD changes following stroke were defined as seed ROIs for FCS analyses using spatially smoothed fMRI data (FWHM = 8 × 8 × 8 mm^3^). For each subject, the correlation coefficient between the mean time series of each seed ROI and that of each voxel in the whole brain was computed and transformed into a *z*-value to improve normality. Subsequently, individuals’ *z*-values were entered into a random effects one-sample *t*-test to identify brain regions exhibiting significant correlations with a given seed ROI. Multiple comparisons were corrected using the family-wise error (FWE) method (*P* < 0.05). A mask that consisted of brain areas with significant FCS with the seed ROI was generated and applied in inter-group FCS comparisons.

### Gray Matter Volume (GMV) Calculation

All structural MR images were carefully examined. Two sets of structural images from two patients were excluded from the GMV analysis due to bad image quality. The structural images were segmented into GM, white matter and cerebrospinal fluid using the standard unified segmentation model of SPM8. Following segmentation, GM population templates were generated from the entire image dataset using diffeomorphic anatomical registration through the exponentiated Lie algebra (DARTEL) technique (Ashburner, 2007). After an initial affine registration of the DARTEL GM template to the tissue probability map in the Montreal Neurological Institute (MNI) space^2^, GM images were nonlinearly warped to the DARTEL template in the MNI space with a resolution of 1.5 × 1.5 × 1.5 mm^3^ (as recommended for the DARTEL procedure). The GMV of each voxel was obtained by multiplying the GM concentration map by the non-linear determinants derived during spatial normalization. Finally, to compensate for residual anatomical differences across subjects, the GMV images were smoothed with a Gaussian kernel of 8 × 8 × 8 mm^3^ FWHM. In effect, the regional GMV represents a normalized GMV after removing the confounding effects of variance in individual brain sizes. After spatial pre-processing, the normalized, modulated, and smoothed GMV maps were used for statistical analysis.

### Statistical Analysis

A mixed-effects model was employed to characterize dynamic changes in the FCD over time in stroke patients. A random intercept term was used to account for the correlation due to repeated measurements within a single patient (Gibbons et al., 1988). This model allowed us to make the utmost use of all available data for each patient, even if some time points were missing. Each patient was assumed to possess a common slope (fixed effect) with only the intercepts allowed to vary (random effect). The model was specified by the following equation:

(1) $$Y_{ij}\;=\;u+b_{i}+X_{ij}\beta_{1}+X_{ij}{}^{2}\beta_{2}+\varepsilon_{ij},\;i\;=\;1,2,\ldots ,N,$$

where *Y_ij_* is the FCD of each voxel from the *j*th scan of the *i*th patient (*i ≦ 8, j≦ 5*); *u* is the intercept term that is common to all subjects; *b_i_* is a random intercept, which allows a unique intercept for each patient; *X_ij_* is the time interval (i.e., days after stroke); *β*_1_ is the scalar of fixed effect; *β*_2_ is the quadratic term;* N* is the number of subjects; and ε_ij_ is the residual error of the model. This model simultaneously considered both the linear (*β*_1_) and nonlinear (*β*_2_) changes; however, we only focused on linear changes to reduce the complexity of our analysis. Model parameters were estimated by the restricted maximum likelihood method. Correction for multiple comparisons was performed using a Monte Carlo simulation with a corrected threshold of *P* < 0.05 and a cluster size of at least 43 voxels. The cluster size threshold was determined by the AlphaSim program in the AFNI Software (parameters: single voxel *P* = 0.01, 5000 simulations, FWHM = 8 mm^3^, cluster connection radius = 5 mm)^3^. This mixed-effects model was also used to investigate the dynamic changes in FCS for regions with significant changes in FCD following stroke. We also used this model to test relations between connectivity changes and changes in GMV, MI and NIHSS scores in stroke patients. In this model, each *X_ij_* is a normalized neurological score (calculated by subtracting the subject-specific mean value from the score for each session) from the *j*th time point of the *i*th patient post-stroke. Differences in functional (FCD and FCS) and clinical (MI and NIHSS) measures among patients at different time points and healthy subjects were compared using the SPSS (Statistical Package for the Social Sciences version 18.0), with the significance level set to *P* < 0.05.

## Results

### Demographic and Clinical Data

**Figure 1:**
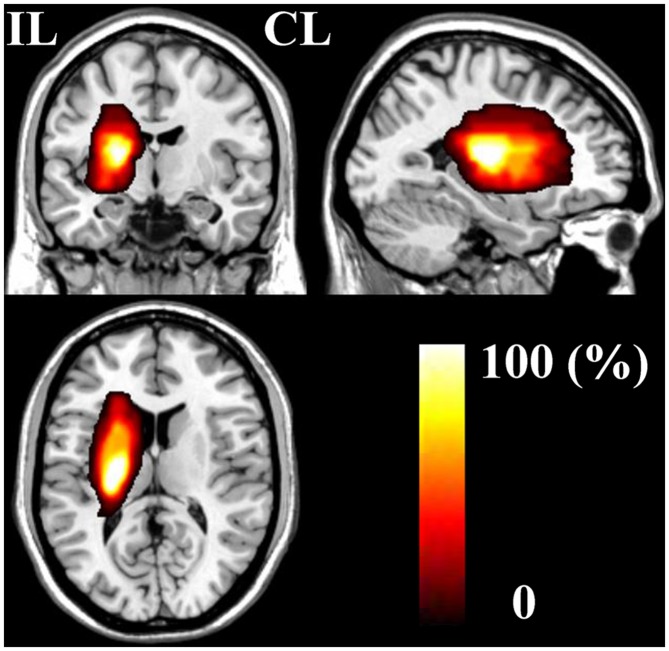
**The stroke patient lesion incidence map.** CL, contralesional; IL, ipsilesional.

**Figure 2:**
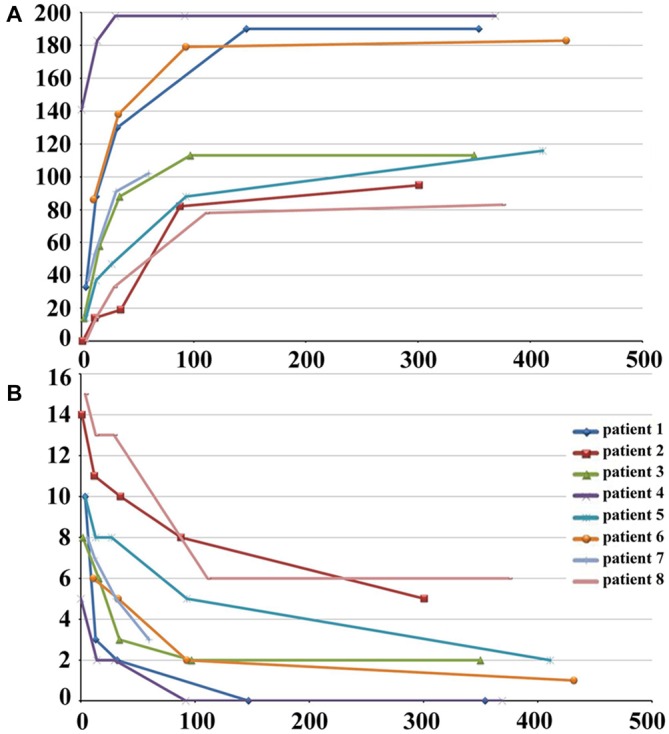
**Post-stroke recovery curves of stroke patients.** The *x*-axis denotes days after stroke onset; *y*-axis denotes MI **(A)** and NIHSS scores **(B)**. MI, Motricity Index; NIHSS, National Institutes of Health Stroke Scale.

The demographic and clinical characteristics of stroke patients are listed in Table 1. The time intervals (mean ± standard deviation) between stroke onset and each session were 3.0 ± 2.1, 13.0 ± 1.5, 31.4 ± 2.7, 97.6 ± 24.5, and 370.3 ± 42.8 days. Six patients completed five sessions and the other two patients finished four sessions. The lesion incidence map for the stroke patients is shown in Figure 1. The lesion volume was 11.5 ± 8.5 ml. Based on the MI and NIHSS scores, all patients exhibited significant recovery (*P* < 0.001), and the recovery curves of these stroke patients are shown in Figure 2. No significant correlations (*P* > 0.05) were observed between lesion volumes obtained at the first time point and neurological examinations (normalized MI and NIHSS) at any time points, suggesting that the lesion volume cannot predict the clinical status of stroke patients.

### FCD Changes in Sensorimotor Regions

**Figure 3:**
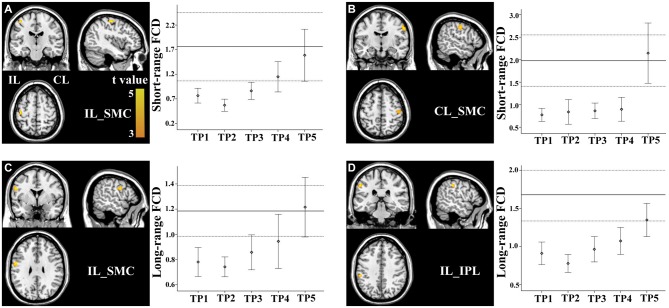
**Linear changes in functional connectivity density (FCD) in the sensorimotor regions in stroke patients.** The ipsilesional **(A)** and contralesional **(B)** SMCs exhibit linearly increased short-range FCD. The ipsilesional SMC **(C)** and inferior parietal lobule (IPL) **(D)** exhibit linear increases in long-range FCD. The solid and dashed lines demonstrate the mean and standard error of the FCD in normal controls, respectively. Error bars illustrate the standard error. CL, contralesional; FCD, functional connectivity density; IL, ipsilesional; SMC, sensorimotor cortex; TP, time points.

Brain regions exhibiting linear changes in FCD following stroke are listed in Table 2. The sensorimotor cortex (SMC) bilaterally exhibited a linear increase in short-range FCD following stroke: the short-range FCD decreased initially (within 1–2 weeks), as compared to controls (*P* < 0.05), remaining at lower levels for months and finally returned to normal levels (Figures 3A,B). The ipsilesional SMC and inferior parietal lobule (IPL) exhibited linearly increased long-range FCD following stroke: the long-range FCD decreased initially, remaining at lower levels for months and finally returned to normal levels (Figures 3C,D).

**Table 2:** **Linear FCD changes after stroke (*P* < 0.05, corrected) and correlations between functional indices and clinical parameters**.

FCD	Brain regions	BAs	Cluster size	MNI coordinates	Peak	MI		NIHSS	
			(voxels)	(*x, y, z*)	*t* values	*t* value	*P*	*t* value	*P*
Short-range FCD	IL_SMC	3, 4	52	−45, −24, 60	3.47	3.379	0.002	−2.432	0.022
Short-range FCD	CL_SMC	3, 4, 6	249	60, −9, 39	4.44	2.249	0.033	−2.176	0.038
Long-range FCD	IL_SMC	6	44	−54, 0, 30	4.22	2.913	0.007	−2.321	0.028
Long-range FCD	IL_IPL	40	47	−54, −30, 42	4.24	2.941	0.007	−2.937	0.007
Long-range FCD	IL DLPFC	10	68	−15, 45, 15	−4.09	−3.243	0.003	3.055	0.005
Long-range FCD	CL_DLPFC	10	70	24, 54, 21	−4.37	−3.086	0.005	4.147	<0.001
Long-range FCD	CL_TC	20, 21	187	54, −24, −30	−4.46	−5.330	<0.001	5.087	<0.001

*BA, Brodmann area; CC, calcarine cortex; CL, contralesional; DLPFC, dorsolateral prefrontal cortex; FCD, functional connectivity density; IL, ipsilesional; IPL, inferior parietal lobule; ITC, inferior temporal cortex; MNI, Montreal Neurological Institute; SMC, sensorimotor cortex; TC, temporal cortex*.

### FCD Changes in Non-Sensorimotor Regions

**Figure 4:**
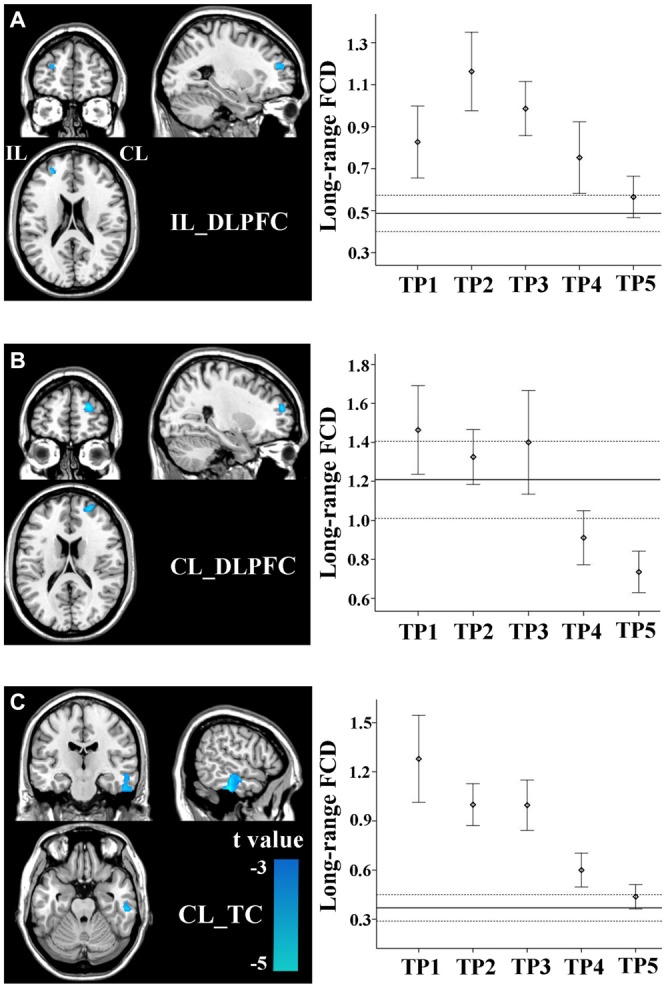
**Linear changes in FCD of the non-sensorimotor regions in stroke patients.** The ipsilesional DLPFC **(A)**, contralesional DLPFC **(B)**, and contralesional temporal cortex (TC) **(C)** exhibit linear decreases in long-range FCD after stroke. The solid and dashed lines demonstrate the mean and standard error of the FCD in normal controls, respectively. Error bars illustrate the standard error. CL, contralesional; DLPFC, dorsolateral prefrontal cortex; FCD, functional connectivity density; IL, ipsilesional; TC, temporal cortex; TP, time points.

Stroke patients also exhibited linear decrease in long-range FCD in several non-sensorimotor regions, including the bilateral dorsolateral prefrontal cortices (DLPFCs) and contralesional anterior temporal cortex (TC). ROI-based comparisons demonstrated that the long-range FCD of these regions increased initially (within 1–2 weeks), as compared to controls (*P* < 0.05), remaining at higher levels for weeks or months and finally returned to normal levels by 1 year post-stroke (Figures 4A–C).

### FCS Changes in Sensorimotor Regions

**Figure 5:**
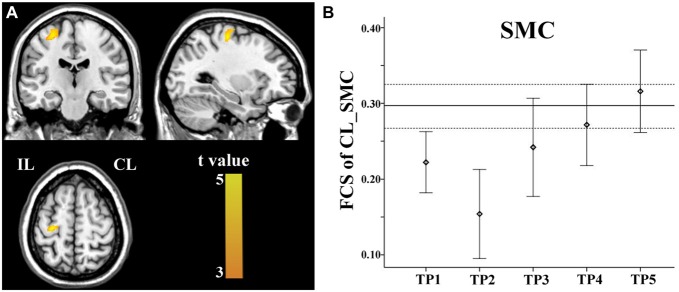
**Linear increase in FCS between the contralesional SMC and the ipsilesional SMC.** The ipsilesional SMC **(A)** exhibit linear increases in FCS with the contralesional SMC. The solid and dashed lines **(B)** demonstrate the mean and standard error of the FCS in normal controls, respectively. Error bars illustrate the standard error. CL, contralesional; FCS, functional connectivity strength; IL, ipsilesional; SMC, sensorimotor cortex; TP, time points.

Brain regions exhibiting linear changes in FCS following stroke are shown in Table 3. Stroke patients exhibited linear increase in FCS between the contralesional SMC and ipsilesional SMC (Figure 5A). Compared to healthy controls, stroke patients exhibited decreased FCS in the first 2 weeks after stroke, followed by increases in FCS towards normal levels (Figure 5B).

**Table 3:** **Linear FCS changes after stroke (*P* < 0.05, corrected) and correlations between FCS strengths and clinical measures**.

Seed regions	Connected regions	BAs	Cluster size	MNI coordinates	Peak	MI		NIHSS	
			(voxels)	(*x*, *y*, *z*)	*t* values	*t* value	*P*	*t* value	*P*
CL_SMC	IL_SMC	4, 6	78	−30, −21, 60	3.66	3.148	0.004	−2.302	0.029
CL_TC	ACC	32	50	15, 39, 12	−4.80	−3.500	0.002	4.056	<0.001
	IL_DLPFC	10	69	−21, 63, 12	−4.05	−2.395	0.024	2.715	0.011
	CL_Cau	–	51	9, 9, 0	−5.06	−2.838	0.008	3.463	0.002
	CL_FG	19, 37	67	45, −66, −18	−3.60	−2.888	0.007	3.271	0.003
	CL_OFC	47	90	33, 27, −18	−3.72	−2.912	0.007	3.133	0.004
	IL_OFC	47	47	−45, 33, −15	−4.43	−3.510	0.002	2.807	0.009
	BL_CC	17	84	3, −60, 9	−4.27	−2.653	0.013	3.359	0.002
	CL_AG	39, 40	143	60, −51, 24	−3.85	−2.509	0.018	3.166	0.004
IL_DLPFC	MPFC	9	90	12, 57, 36	−3.67	−2.688	0.012	2.758	0.010

*ACC, anterior cingutate cortex; AG, angular gyrus; BA, Brodmann area; BL, bilateral; Cau, caudate nucleus; CC, calcarine cortex; CL, contralesional; DLPFC, dorsolateral prefrontal cortex; FCD, functional connectivity density; FCS, functional connectivity strength; FG, fusiform gyrus; IL, ipsilesional; ITC, inferior temporal cortex; MNI, Montreal Neurological Institute; MPFC, medial prefrontal cortex; OFC, orbitofrontal cortex; SMC, sensorimotor cortex; TC, temporal cortex*.

### FCS Changes in Non-Sensorimotor Regions

**Figure 6:**
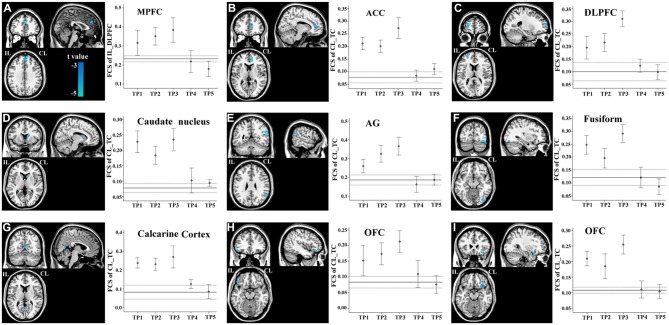
**Brain regions with linear decrease in FCS with the ipsilesional DLPFC and contralesional TC. A–I** show the various brain regions with linear decrease in FCS with the ipsilesional DLPFC and contralesional TC; and the solid and dashed lines demonstrate the mean and standard error of the FCS in normal controls, respectively. Error bars illustrate the standard error. ACC, anterior cingulate cortex; AG, angular gyrus; CL, contralesional; DLPFC, dorsolateral prefrontal cortex; FCS, functional connectivity strength; IL, ipsilesional; MPFC, medial prefrontal cortex; OFC, orbitofrontal cortex; TC, temporal cortex; TP, time points.

FCS changes were also observed in non-sensorimotor regions in stroke patients. The ipsilesional DLPFC exhibited a linear decrease in FCS with the contralesional medial prefrontal cortex (Figure 6A). The contralesional anterior TC exhibited a linear decrease in FCS with the anterior cingulate cortex, ipsilesional DLPFC, contralesional caudate nucleus, angular gyrus and fusiform gyrus, and calcarine cortex and orbitofrontal cortex bilaterally (Figures 6B–I). The FCS increased initially, as compared to controls (*P* < 0.05), remaining at higher levels for the first month, and finally returned to normal levels (Figure 6).

### Relations Between Connectivity and Structural Changes

Correlations between connectivity (FCD and FCS) and GMV changes in these hub regions following stroke were also investigated using the mixed-effects model. The short-range FCD of the ipsilesional SMC (*t* = −2.636, *P* = 0.014) and long-range FCD of the ipsilesional SMC (*t* = −2.800, *P* = 0.010) and IPL (*t* = −2.451, *P* = 0.021) were negatively correlated with GMV changes in the corresponding regions in stroke patients. No significant correlations were found between connectivity and structural changes in any other regions that showed significant changes in the FCD.

### Associations Between Connectivity and Clinical Scores

The mixed-effects model was applied to explore relations between connectivity (FCD and FCS) and clinical scores (MI and NIHSS) after stroke. The FCD and FCS in most of these hub regions were significantly (*P* < 0.05) correlated with both clinical scores during stroke recovery (Tables 2, 3).

### Functional Connectivity Pattern of Each Hub Region

**Figure 7:**
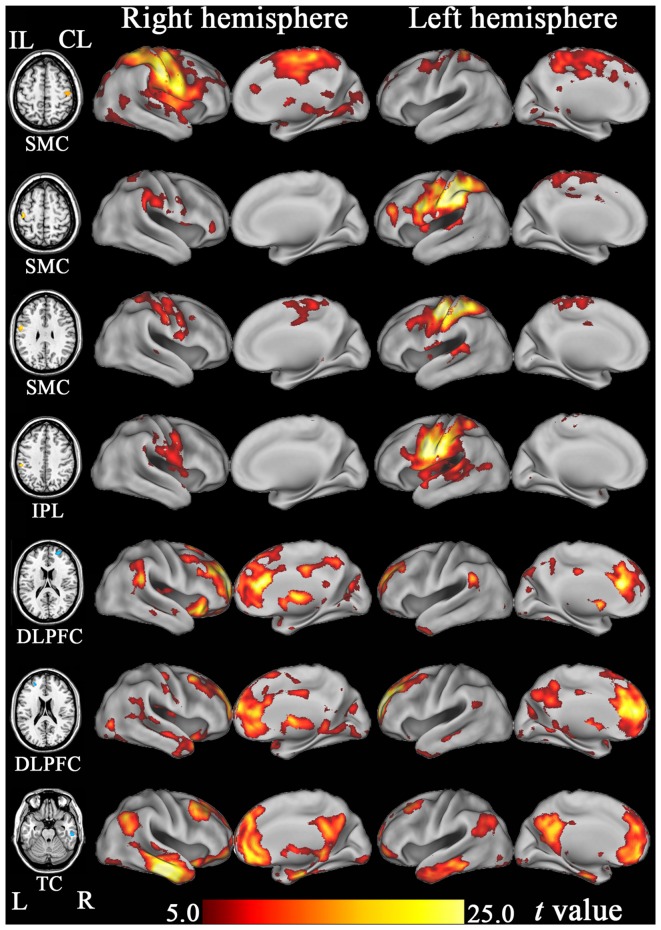
**The resting-state functional connectivity maps of hub regions that show significant FCD changes after stroke.** DLPFC, dorsolateral prefrontal cortex; FCD, functional connectivity density; IPL, inferior parietal lobule; ITC, inferior temporal cortex; SMC, sensorimotor cortex; TC, temporal cortex.

Based on the functional connectivity patterns, we can infer the possible function of each hub region. We found that the SMCs and IPL were similarly connected to brain regions subserving for sensorimotor processing (Figure 7). As a putative region for cognitive control, the DLPFC mainly connected to other brain regions associated with cognitive control, such as the dorsal anterior cingulate cortex and nearby prefrontal regions (Figure 7). The anterior TC primarily connected to brain regions of the default-mode network, including the medial prefrontal cortex, posterior cingulate cortex, and lateral parietal cortex (Figure 7). The default-mode network is a canonical resting-state functional network of the brain and has been associated with cognitive processing (Mantini and Vanduffel, 2013).

## Discussion

In this longitudinal study, we combined FCD and FCS to investigate the dynamic connectivity reorganization of the brain after subcortical stroke in the motor pathways. Besides the sensorimotor regions, the cognitive regions also exhibited significant dynamic connectivity changes following stroke. However, the temporally-evolving patterns of these two kinds of regions were largely different: FCD and FCS in the sensorimotor regions decreased initially, as compared to controls, remaining at lower levels for months, and finally returned to normal levels; however, connectivity measures in the cognitive regions increased initially, remaining at higher levels for months, and finally returned to normal levels. These network-specific connectivity reorganization patterns suggest that the sensorimotor and cognitive regions may reorganize themselves in different temporal dynamics to facilitate motor recovery after subcortical stroke.

### Dynamic Connectivity Reorganization in the SMN

This is the first attempt to use voxel-wise FCD analysis to identify brain regions with significant connectivity changes following stroke. In the sensorimotor regions, FCD decreased initially, remaining at lower levels for months, and finally returned to normal levels. The similar temporally-evolving patterns in the sensorimotor regions were also confirmed by our and previous longitudinal FCS analyses in the SMN in stroke patients (Wang et al., 2010; Xu et al., 2014). The consistency across these studies suggests that these connectivity changes represent a general evolutionary pattern in the SMN. We also found that connectivity restoration in the sensorimotor regions bilaterally was correlated with motor recovery after stroke. Although the relationship between FCS restoration in the ipsilesional SMC and motor recovery has been previously confirmed (Park et al., 2011; Zhang et al., 2014), our finding of the correlation between FCS restoration in the contralesional SMC and motor recovery may support that functional changes in the contralesional SMC may contribute to motor recovery after stroke. Although the neural mechanisms underlying the initial decrease in the functional connectivity of the SMN remain unclear, this decrease may be related to transient diaschisis (Andrews, 1991). The transient breakdown of harmonious interaction between the SMN regions after ischaemic injury of the motor pathway may underlie the initial connectivity impairment in these regions. In support of our findings, dynamic evolution in the FCS between the bilateral SMC has been well documented in rats subjected to stroke and human stroke patients (van Meer et al., 2012; Rehme and Grefkes, 2013), and this FCS change has been associated with motor recovery after stroke. We also found negative correlations between structural and functional changes in SMN regions following stroke, suggesting that the functional changes observed in the SMN at least partly reflect a compensation for underlying structural damage.

### Dynamic Functional Reorganization in the CPN

In stroke patients, the FCD and FCS of the bilateral DLPFC and the contralesional TC exhibited a different temporally-evolving pattern compared to that seen for the connectivity changes of the SMN. These measures increased significantly immediately after stroke onset, remained at an elevated level for weeks or months and then decreased to normal levels by 1 year after stroke. Functional connectivity patterns of these regions suggest that the DLPFC is a region involved in cognitive control, whereas, the anterior TC is a component of the default-mode network and is also involved in cognitive processing (Mantini and Vanduffel, 2013). The dynamic connectivity patterns in the cognitive-related region are consistent with previous longitudinal studies on motor-evoked activation in subcortical stroke patients. These studies have displayed that activation increases initially and then returns towards normal levels in several cognitive-related regions, including the prefrontal cortex, anterior cingulate cortex, TC, and parietal cortex (Marshall et al., 2000; Ward et al., 2003). The involvement of the cognition-related regions in motor recovery following stroke is also reported in subcortical stroke patients with good motor outcomes. During a motor imagery task, these patients exhibit significantly enhanced effective connectivity between the prefrontal cortex and motor areas, and this increase in connectivity correlates with motor outcome (Sharma et al., 2009). Moreover, a recent cross-sectional study has revealed resting-state connectivity changes within and between several cognitive-related networks in chronic subcortical stroke patients (Wang et al., 2014). As the first investigation on the longitudinal connectivity changes in cognitive regions following stroke, our findings may indicate a neural mechanism of cognitive compensation for motor deficits.

### An Integrated View for Neural Mechanisms for Stroke Recovery

During the stroke recovery process, we found completely different evolutionary patterns of connectivity reorganization in sensorimotor and cognitive regions. These phenomena cannot be understood in isolation; instead, they should be interpreted in an interactive way. Shortly after stroke onset, the function of the SMN hubs is compromised, as suggested by the decrease in connectivity. At the same time, the connectivity in the cognitive hubs increases in response to increased mental and physical effort (Schmidt et al., 2012). The increased connectivity between these cognitive hubs may generate stronger motor control signals to the SMN hubs, where movement programs are encoded and executed. This process is cycled repeatedly during motor recovery to re-establish the smooth execution of motor tasks in stroke patients, and this repeated effort may account for the subsequent recovery of motor function. In the chronic stage of stroke, as motor skills are reacquired and resting-state functional properties recover, a lack of demand for cognitive support may explain subsequent decreases observed in the connectivity of the cognitive hubs. However, it should be noted that the reciprocal relation between these hub regions may, in fact, be rather complex. Several limitations should be mentioned in this study. First, the sample size was rather small (8 patients and 10 controls), which may make our study to be statistically underpowered. Second, we did not assess the cognitive functions, which may help to clarify the functional role of our findings. Future studies are needed to validate these interpretations of our results.

In conclusion, this study identifies the dynamic connectivity changes of the brain during the stroke recovery process. The temporally-evolving patterns in connectivity of the sensorimotor hubs were different from those of cognitive hubs. The network-specific functional reorganization patterns we observed are consistent with the hypothesis of cognitive compensation for motor deficits. We suggest that future studies of motor recovery after stroke should consider interactions among multiple functional networks rather than the SMN alone.

## Author Contributions

HL, TT and CY designed the experiment and wrote the protocol and the draft of manuscript text. WQ, HL and TT performed image processing and statistical analyses. WQ and KL collected the magnetic resonance imaging (MRI) data.

## Funding

This study was supported by grants from the Natural Science Foundation of China (Nos. 81401379, 81425013, 91332113 and 81271551) and the Tianjin Key Technology R&D Program (14ZCZDSY00018).

## Conflict of Interest Statement

The authors declare that the research was conducted in the absence of any commercial or financial relationships that could be construed as a potential conflict of interest.

## Footnotes

^1^http://www.fil.ion.ucl.ac.uk/spm

^2^http://www.mni.mcgill.ca/

^3^http://afni.nimh.nih.gov/
